# Defensive Mechanisms of *Mikania micrantha* Likely Enhance Its Invasiveness as One of the World’s Worst Alien Species

**DOI:** 10.3390/plants14020269

**Published:** 2025-01-18

**Authors:** David R. Clements, Hisashi Kato-Noguchi

**Affiliations:** 1 Department of Biology, Trinity Western University, 22500 University Drive, Langley, BC V2Y 1Y1, Canada; clements@twu.ca; 2 Department of Applied Biological Science, Faculty of Agriculture, Kagawa University, Miki, Kita 761-0795, Kagawa, Japan

**Keywords:** allelochemicals, epigenetic variation, invasive species, genetic variation, natural enemies, nematodes, pathogens, vegetative reproduction

## Abstract

*Mikania micrantha* Kunth is native to tropical America and has invaded tropical and subtropical Asia and numerous Pacific Islands. It forms dense thickets and reduces native species diversity and populations in its introduced range. This invasive vine also seriously impacts many agricultural crops and is listed as one of the world’s 100 worst invasive alien species. Its life history characteristics, such as the production of large numbers of wind-dispersed seeds, vegetative reproduction, rapid growth, and genetic diversity all contribute to its invasiveness. In this review, we focus on how mechanisms to defend against its natural enemies boost the invasiveness of *M. micrantha*. It possesses potent defenses against natural enemies such as pathogenic fungi, herbivorous insects, and parasitic nematodes, and exhibits allelopathic potential against plant competitors. These defensive abilities, in concert with its formidable life history characteristics, contribute to the invasiveness of *M. micrantha*, potentially leading to further naturalization. Several other reviews have summarized the biology and management of the species, but ours is the first review to focus on how the defensive mechanisms of *M. micrantha* likely enhance its invasiveness. Relatively little is known about the array of defensive capabilities of *M. micrantha*; therefore, there is considerable scope for further research on its chemical defenses.

## 1. Introduction

*Mikania micrantha*, a vine in the Asteraceae native Central and South America commonly known as mile-a-minute, has become widespread in Asia and the Pacific Islands and is listed among the world’s top 10 world’s worst weeds, negatively impacting many crops and natural ecosystems [1,2], as detailed in Section 1.3 and Section 1.4. Commonly called mile-a-minute, true to its name it grows rapidly, with recorded growth rates as high as 20 cm per day under ideal conditions [3]. It also possesses many other adaptations, which enable it to suppress and compete with other vegetation, such as adventitious rooting from the nodes in the vines [4,5], the release of allelochemicals [6,7] and a generally high degree of morphological and physiological plasticity [4,8].

### 1.1. Classification and Description

*Mikania micrantha* Kunth, commonly known as mile-a-minute, is a perennial vine in the Asteraceae family. It falls within the Eupatoriea tribe, a neotropical grouping of asters with whitish flowers containing only disk florets, where *Mikania* is the largest genus consisting of about 430 species [9], half of which are found in Brazil [10].

Synonyms for *M. micrantha* include: *Eupatorium denticulatum* Vahl, *Eupatorium orinocense* (Kunth) M. Gómez, *Kleinia alata* Meyer, *Mikania alata* (*Meyer)* D.C., *Mikania cissampelina* DC., *Mikania denticulate* (Vahl) Willd., *M. glechomaefolia* Sch.-Bip. ex Bak. Mart. Fl., *M. orinocensis* Kunth, *M. sinuata* Rusby, *M. subcrenata* Hook. & Arn., *M. subcymosa* Gard., *M. umbellifera* Gard., *M. variabilis* Meyen & Walp., and *Willoughbya micrantha* (Kunth) Rusby [11]. *Mikania micrantha* is referred to as *M. micrantha* H.B.K. in some manuscripts rather than *M. micrantha* Kunth. Some confusion exists among *M. micrantha*, *M. cordata* (Burm.f.) B. L. Rob., *M. cordifolia* (L.f.) Willd, and *M. scandens* (L.) Willd [1,11,12].

In addition to mile-a-minute, *M. micrantha* has many different common names such as bittervine, hemp vine, American rope, or Chinese creeper [13], with the most colorful moniker perhaps being “plant killer”, a nickname in China referring to its ability to smother other plants [11,13,14].

**Figure 1:**
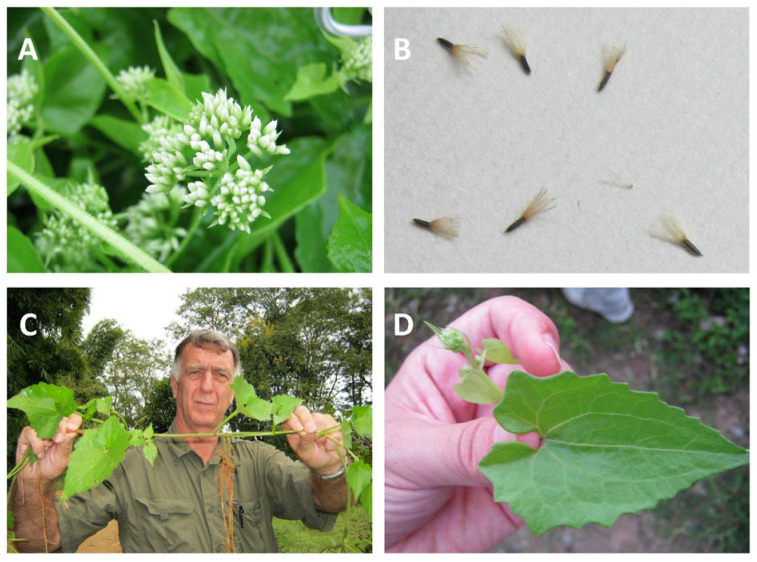
Plant anatomy of *Mikania micrantha*. (**A**) flowering cluster showing distinctive 4.5–60.0 mm white flowers, (**B**) seeds with pappuses (1.5 mm long), (**C**) adventitious roots growing from a vine (with Joseph DiTomaso, University of California), (**D**) heart-shaped leaf structure (4 to 13 cm in length). Photos by David R. Clements taken in Yunnan Province, China.

*Mikania micrantha* is readily distinguished from other *Mikania* species by its distinct leaf and flowering structures (Figure 1). It has two different growth forms, depending on the habitat where it is growing: prostrate growing where there is low-growing vegetation and a climbing habit in the presence of tall, woody vegetation. Its 4–13 cm long leaves are opposite, with heart-shaped bases and pointed apices, often with serrated margins but occasionally more entire. Clusters of flowerheads are formed, with the white or greenish fragrant flowers being 4.5–6.0 mm long. Flower bracts are 3–4 mm long for *M. micrantha*, shorter than the 5–6 mm long typical for *M. cordata*, which also produces larger seeds. In addition, the nodal appendage between the leaf stalks is hairless in *M. micrantha* but pubescent in *M. cordata* [12,15]. Black, linear oblong 1.5 mm seeds are produced with white bristles at the top of the pappus ([1,16] Figure 1B). When growing in its prostrate form, many adventitious roots sprout from the main stem and branch nodes [5]. Whereas the initial roots from seed germination form at the base of the new stem, adventitious roots form above the growing stem and branch nodes as the vine elongates (Figure 1C). As the vines spread, the adventitious roots can establish new points of contact with the soil and increase overall growth [5].

### 1.2. Invasion History

*Mikania micrantha* first reached the Old World in 1884 as part of the living plant collection at the Hong Kong Zoological and Botanical gardens; after escaping the gardens it became naturalized in Hong Kong by 1919 [17,18]. Because growing conditions in Asia closely matched its native neotropical habitat, many more invasions in the region followed. It reached mainland China by 1910 when it was reported to be established in Guangdong Province [14]. It has since spread through China’s southern provinces [19,20] and was naturalized in Taiwan by 1986 [21].

The first report of *M. micrantha* being naturalized in India was in 1918 and is said to have been reintroduced deliberately in World War II for camouflage purposes [15]. It reached Indonesia by 1949 and Nepal by the 1960s, with some introductions facilitated by its use as a cover crop [22,23]. Its deliberate introduction to Malaysia in the 1950s was reportedly to provide ground cover for rubber plantations [11].

In the Pacific Islands, the first report of *M. micrantha* establishment is dated 1907 [24]. It reached Samoa, American Samoa, Vanuatu, and Niue by the mid-19th century [13]. Naturalized populations appeared on other Pacific Islands later: Papua New Guinea in 1951, Guam in 1963, Tonga in 1979, the Solomon Islands in 1988, the Cook Islands in 1991, the Northern Mariana Islands in the 1990s, the federated States of Micronesia in 2000, and Kiribati in 2012 [7,13]. It was first reported in Australia near Brisbane in 1998 [25].

In 2010, the only recorded infestation of *M. micrantha* in the continental United States was reported in Florida in the vicinity of Miami [26]. It threatens to invade Hawai’i because of its favorable habitats and the frequent interceptions of *M. micrantha* seeds on military vehicles transported from Guam [13].

### 1.3. Ecological Impacts

**Figure 2:**
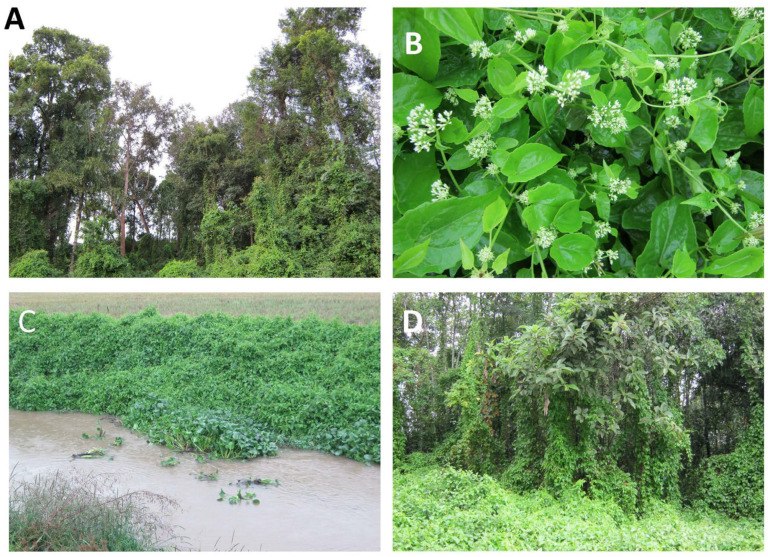
Infestations of *Mikania micrantha* in Yunnan Province, China. (**A**) Forest being overtaken by *M. micrantha* vines, demonstrating the ability of the vines to attain heights of 10 m or more, (**B**) *M. micrantha* in flower, (**C**) *M. micrantha* in a riparian zone on a river, showing potential to spread. (**D**) Trees being smothered by *M. micrantha* vines. Photos by David R. Clements.

*Mikania micrantha* invades a variety of natural systems in its introduced habitat, and is particularly damaging in disturbed forest habitats, forest edges or in riparian zones with shrubs and trees where it can use the vegetation as scaffolding for its climbing habit (Figure 2A,D). Under such conditions the plant can grow as high as 15 m, growing over the canopy and blocking available light to the trees. Closed canopy habitats are not nearly as susceptible to invasion because of the requirement of *M. micrantha* for relatively high light levels. Grasslands with few shrubs or trees do not generally support significant populations of *M. micrantha* [27,28]. Forests with closed canopies and larger trees creating more shade are also less vulnerable [27,29,30]. Disturbed areas with limited shade also allow *M. micrantha* to produce abundant flowers and seeds (Figure 2B). It is also able to flourish in riparian areas because its vegetative spread and hydrochory are facilitated along rivers (Figure 2C). In such areas, it can form thick monospecific mats, inhibiting the growth of competing vegetation [1,14]. Kaur et al. [6] observed 30% less species richness in habitats invaded by *M. micrantha* in the Western Ghats in India.

Where *M. micrantha* forms extensive monocultures in its invasive range, populations of native fauna may be seriously jeopardized. For example, in Nepal’s Chitwan National Park, the plants usually browsed by the endangered one-horned rhinoceros (*Rhinoceros unicornis* L.) threaten these diminishing populations [31]. Another negative impact of burgeoning *M. micrantha* populations in Nepal is how the lack of natural vegetation forces buffalo and wild boar to migrate into crop areas [32]. The one-horned rhinoceros habitat is also threatened in North East India [33]. Other critically endangered fauna in these Indian grasslands threatened by the *M. micrantha* invasion include the pygmy hog (*Porcula salvania* Hodgson), and a charismatic grassland bird, the Bengal florican (*Houbaropsis bengalensis* Gmelin) [33]. Native fauna in heavily invaded habitats in China have also been negatively impacted, such as native rhesus macaque monkey *Macaca mulatta* Zimmerman populations on Neilingding Island, where banana trees, a major food source for the monkeys, were depleted by the invasion [34].

*Mikania micrantha* also impacts soil health. Reduced nitrification and modification of soil pH and organic matter in soil infested by *M. micrantha* has been reported [35]. Li et al. [36] observed that the change in pH resulted in a number of changes, including more aerobic bacteria, less anaerobic bacteria, and more enzyme activity. Overall, it is clear that profound changes to the microbial community take place in areas invaded by *M. micrantha* [36]. Allelopathic chemicals released by *M. micrantha* should be accounted for when developing management strategies, as the allelochemicals may impact the soil microbial community and soil nutrient levels [37]. The soil microfauna represents another ecosystem component that can influence soil health in invaded areas. Sun et al. [38] found that *M. micrantha* could influence nematode feeding rates on bacteria, thereby reducing bacterial processes such as the release of potassium from the soil.

### 1.4. Economic Impacts

As one of the world’s worst weeds [1], the economic impacts of *M. micrantha* are substantial. Just within the Pearl River Delta in China, estimated annual losses due to *M. micrantha* were 8 billion RMB (about $1.2 billion USD) per year in 2017 [39]. In 2011, Zhang et al. [40] estimated annual economic losses on Neilingding Island at between $650,000 and $1,600,000 USD. Substantial economic losses are seen in both agricultural crops and forestry throughout the invaded range in the Asian tropics and subtropics and in the Pacific Islands where it occurs. Because it is generally quite costly and labor intensive to control [41], economic losses due to *M. micrantha* are often calculated based on both lost productivity and the cost of control. Herbicide costs tend to be higher than for other weeds because of the ability of *M. micrantha* to regrow from the roots after spraying [28].

Another factor that can increase the cost of *M. micrantha* is interference with crop harvesting. Some prominent examples of crops where *M. micrantha* interferes with harvesting are oil palms, cacao, tea, sugar cane, and coconuts [1,28,42]. A survey of farmers in Papua New Guinea revealed a heavy toll on farmers relying on manual control of *M. micrantha*, where farmers spent inordinate amounts of time weeding. This severe impact is seen in many developing countries, and in Samoa, some farmers abandoned their landholdings because they could not keep up with *M. micrantha* management [7,28].

Through its climbing habitat and ability to smother tree crops, *M. micrantha* can have severe impacts on plantation trees, including banana (*Musa* spp.), various citrus trees (*Citrus* spp.), cacoa (*Theobroma cacao* L.), coffee (*Coffea arabica* L.), tea (*Camellia sinensis* (L.) Kuntze), teak (*Tectona grandis* L. f.), rubber (*Hevea brasiliensis* (Willd. ex A.Juss.) Müll. Arg.), African oil palm (*Elaeis guineensis* Jacq.), and common bamboo *(Bambusa vulgaris* Schrad.) [7,27]. A yield loss estimate of 42% was reported for tea plantations in India [42]. Another crop that can be heavily impacted by *M. micrantha* is sugar cane (*Saccharum officinarum* L.), with estimates of yield loss in infested fields in China ranging as high as 60 to 70% [43]. In fact, crop losses can approach 100%, where large populations of *M. micrantha* smother the crop [28,44]. A variety of crops may experience reduced germination rates due to allelochemicals from *M. micrantha* [6,45].

Various forest trees with economic importance are impacted by competition with *M. micrantha*. For example, the growth of Indian rosewood (*Dalbergia sissoo* Roxb. ex DC.) and cotton tree (*Bombax ceiba* L.) is curtailed by *M. micrantha* in Nepal [27]. Livestock production can also be reduced in pastures infested by the weed, through decreased productivity of pasture grasses or hepatotoxicity of *M. micrantha* to the animals [13,46].

## 2. Life History Traits

### 2.1. Growth

True to its common name “mile-a-minute”, high growth rates have been measured under field conditions for *M. micrantha*. As mentioned earlier, growth rates as high as 20 cm day^−1^ have been recorded in China [3]. Other growth rates recorded are generally lower but are still very rapid among plant species. In India, growth rates of 8–9 cm day^−1^ were reported [15]. Growth rates of 3.3 cm day^−1^ were measured in Papua New Guinea [28], and 2.7–3.8 cm day^−1^ in Fiji [47]. Through a functional analysis of the *M. micrantha* genome, Liu et al. [4] demonstrated that the rapid growth rate of *M. micrantha* by comparison to other plants is a result of unique physiological characteristics. Increased photosynthetic capacity is achieved through nighttime carbon fixation, which enhances the CO_2_ absorbed during the day and through high stem photosynthetic efficiency that enhanced the photosynthetic output of the leaves [4]. This photosynthetic ability driving rapid stem growth compared to other plants is supported by relatively more genes involved in the photosynthetic pathway and related systems for carbon capture [48]. Liu et al. [4] also showed how metabolites produced by *M. micrantha* could increase N availability through their positive influence on microflora.

Further details of the anatomy and physiology of *M. micrantha* facilitating these rapid growth rates have been recently uncovered. Jiang et al. [49] reported that *M. micrantha* responded to lower light levels by extending internodes and epidermal cells, enabling it to grow longer stems. They traced this response to the regulation of phytohormones and photoprotective substances [49]. It was also shown that *M. micrantha* can compensate for defoliation with increased stem elongation and stem photosynthesis [50]. This increased stem photosynthesis is due to a variety of factors working together, including modifications in the chlorophyll a/b ratio, protein content, stomatal openings, chloroplast morphology, and anthocyanin content [50]. Furthermore, when growing in its prostate form, *M. micrantha* is capable of producing large numbers of adventitious roots, and it was shown by Shen et al. [5] that plants producing more adventitious roots also exhibited higher biomass, stem length, and branch number.

*Mikania micrantha* affects many crop species, particularly those that provide scaffolding for its climbing growth habit. Its rapid growth enables it to completely smother crops or trees, blocking out sunlight, thus reducing growth, flowering, and yield [28,30,32]. The rapid growth of *M. micrantha* and its ability to resprout from stem fragments facilitate its ability to smother vegetation as the vines grow over crops or trees, resulting in reduced growth or mortality of the competing vegetation [12,28,51]. Chen et al. [52], in an in-depth examination of the mechanism by which *M. micrantha* smothers trees and shrubs, discovered that lack of support for upper branches as the *M. micrantha* vine reaches the top of the tree stimulates a turning growth pattern and hormones to release bud dormancy, resulting in the creation of more branches, which tend to smother the treetops.

### 2.2. Reproduction

*Mikania micrantha* undergoes both sexual and asexual reproduction [1,15]. *Mikania micrantha* vines produce 93,000–154,000 flowers/0.25 m^2^ [53]. From this prolific flower production, seed production ranging from 90,000 to 210,000 seeds/m^2^ may be achieved by *M. micrantha* [53,54]. The seeds possess a pappus comprised of 4 mm bristles facilitating long-distance aerial dispersal and also increasing their floatation ability in water [53,55,56]. The pappus also facilitates zoochory, with the seeds becoming attached to animal fur or human clothing [1,56]. Additionally, the pappus enhances water absorption, thus improving germination rates [56]. Genetic studies of *M. micrantha* reveal a high degree of genetic diversity among biotypes and local populations, suggesting sexual reproduction may often be the dominant form of reproduction [57].

Successful reproduction through flowering depends on adequate pollination as the flowers are generally self-incompatible [57]. Insect pollination greatly enhances pollination, as demonstrated by Hong et al. [58], who recorded an average number of seeds per ovule of 0.56 for insect pollinated plants, as compared to 0.0034 for wind pollination and 0.0038 for self-pollination.

Asexual reproduction can occur via the formation of ramets emerging from rosettes, and in certain areas, asexual reproduction from ramets is clearly the more dominant form of reproduction, particularly where flowering or insect pollination is reduced due to unfavorable environmental conditions [54,59]. Other common means by which *M. micrantha* is propagated vegetatively include fragmenting and resprouting or the development of adventitious roots when the stems come into contact with soil [5,28,43,51]. Zhang et al. [60] observed climbing plants exhibited far more reproduction through flowering than prostate plants (85% versus 40%), illustrating the adaptability of *M. micrantha*. Yue et al. [61] demonstrated that flooding with shallow water increased the invasiveness of *M. micrantha*. Seeds are able to germinate below the water’s surface and emerge from a depth of 6 cm [61]. Plant growth parameters such as root structure and vine length and growth were shown to be enhanced in shallow water or wet environment [61].

The generally prolific reproductive abilities of *M. micrantha* are greatly reduced by shading or competition with other plants, which inevitably translates to reduced sexual and asexual reproduction. Shen et al. [62] found that sweet potato, *Ipomoea batatas* (L.) Lam. tended to outcompete *M. micrantha* when the two plants were grown together, and this impact of sweet potato was enhanced by fertilizer application. When grown with both *I. batatas* and hyacinth bean, *Lablab purpureus* (L.) Sweet, *M*. *micrantha* was more suppressed than by either crop alone [63]. *Mikania micrantha* is likewise affected by competition with itself, as Huang et al. [64] found that increased planting densities of *M. micrantha* led to the production of fewer and larger seeds with reduced dispersal capabilities.

### 2.3. Adaptations to Habitat

In its native range in the neotropics, *M. micrantha* is generally found in moist habitats that receive relatively high rainfall levels, such as forest edges, riparian areas, and lowland areas with relatively open canopies, ranging to altitudes of 3000 m [1,65,66]. In its native range, it does not tend to be or form large monocultures as seen in its invasive range ([67], R. Zenni pers. Comm.).

Certain crops are more vulnerable to *M. micrantha*, especially tree crops, orchards, and other types of plantations where *M. micrantha* can take advantage its climbing habit [27,28,30,68]. Habitats with relatively high light intensity, moisture, and fertility, tend to support the highest growth rates for *M. micrantha* [69,70]. More heavily shaded habitats, such as native forests, are less favorable for the growth of *M. micrantha* [1,70].

### 2.4. Genetic Variation and Adaptability

In its native range, *M. micrantha* may be diploid (2n = 2x = 36), aneuploid (2n = 2x = 42), or tetraploid (2n = 2x = 72) [10,66]. *Mikania micrantha* flowers are self-incompatible and thus is it an obligate outcrossing species [57]. Considerable variation occurs among populations in the introduced range, as indicated by variable susceptibility to *Puccinia spegazzinii* de Toni, a pathogen used in biological control. These differences are evident in comparing populations in India, with populations from Papua New Guinea or Fiji with Indian *M. micrantha* populations more susceptible to *P. spegazzinii* collected in Peru or Trinidad [71,72].

Genetic variation in *M. micrantha* has been studied extensively in the past decade. The *M. micrantha* genome was published by Liu et al. [4] in 2020, which revealed important insights into what makes *M. micrantha* so successful as an invasive plant. The structure of the genome itself is of significance, with the whole genome having been duplicated at some point. The genome also features long terminal repeat retrotransposons, with 80% of these relatively recent (the last million years). As mentioned in Section 2.1, the analysis by Liu et al. [4] demonstrated the unique photosynthesis process and enhanced nitrogen assimilation achieved by *M. micrantha*. Taken together, the various properties of the *M. micrantha* genome provide a strong basis for its adaptability [4]. Stress responses by *M. micrantha* were studied at the molecular level through transcriptome analysis by Ruan et al. [73]. They found that the expression of antioxidant genes was highest in roots, stems and leaves as compared to other organs, indicating the roots, stem and leaves are well-equipped to respond to stresses.

Other recent work has focused on the population genetic structure [17,74,75,76] and epigenetics [77,78] of *M. micrantha*. Much of what we know about the population genetics of *M. micrantha* has come from genetic research in China where *M. micrantha* is a serious pest [44]. A genetic analysis of range expansion in southern China revealed that there was an active selection for seed traits favoring higher levels of dispersal, such as plume loading, seed mass, and pappus radius [17]. Through a genetic analysis of Chinese *M. micrantha* populations Ji et al. [74] traced the history of its spread in China since it first arrived there via Yunnan Province. As other studies have found, there was considerable genetic diversity locally, signifying high levels of sexual reproduction, with 83% of the variation within populations and only 18% among populations. They also found evidence for multiple introductions, with the high level of diversity created by high gene flow levels enabling the plant to adapt to local conditions [74]. Similarly, another analysis of Chinese *M. micrantha* populations identified high levels of diversity within populations and multiple invasion events [75]. Ruan et al. [75] were also able to show that important factors affecting the invasiveness of *M. micrantha* in China included soil composition, temperature, and precipitation, which, combined with high levels of gene flow, make the plant highly adaptable.

The population genetic structure analysis by Banerjee et al. [76] included populations from Mainland China, Hong Kong, Macau, Taiwan, India, Bangladesh, Vanuatu, Sri Lanka, Malaysia, Indonesia, the Philippines, Singapore, Thailand, and Japan. Their analysis involved 46 populations and 1052 individuals [76]. Across Asia, the same high level of genetic diversity is seen that is evident within China [76]. The genetic variation among populations was likewise generally greater than that within populations, with evidence of population bottlenecks in founder populations, but at the same time, high rates of gene flow facilitated the bolstering of diversity in regions with variable environments requiring a high degree of adaptability [76].

In addition to genetic variation created by gene flow and outcrossing, it is evident that epigenetics also supports the adaptability of *M. micrantha* [77,78]. Shen et al. [77] indicated that *M. micrantha* exhibited greater epigenetic than genetic variation. Su et al. [78] found that both transposable elements (TE) contributed greatly to population variability in the populations they studied in China. In just 21 populations, they were able to identify 59 genetic and 86 epigenetic TE loci [78]. TE increases variation in *M. micrantha* and other angiosperms by regulating gene expression and rearranging genomes, mutations, epigenetics, and phenotypic variation [79,80]. Su et al. [78] showed that the genetic and epigenetic loci were correlated with various environmental variables, indicating the potential to adapt to local conditions. Shen et al. [77] pointed out that this epigenetic variation may help *M. micrantha* cope with genetic bottlenecks. They also posited that because much of the epigenetic variation is linked to climatic factors, the northward expansion of *M. micrantha* predicted due to climate change will be enhanced by epigenetic-mediated variability [77].

Despite the evidence for diverse genotypes fueled by reproduction by seed, the high capacity for *M. micrantha* to spread asexually cannot be discounted and clearly helps the plant to adapt to particular circumstances. For example, *M. micrantha* in its prostrate growth form was found to be more likely to reproduce asexually than when growing as a climber (60% vs. 15%) [40]. In any case, the nature of *M. micrantha* as a vine that can form connections with either the soil (Figure 1C) or other vegetation as it grows enables it to adapt to a great variety of habitats, with obvious implications for enhancing its already potent defenses against natural enemies as discussed in the next section.

## 3. Defenses Against Natural Enemies and Allelopathy

### 3.1. Natural Enemies in the Introduced Range of M. micrantha

The population of a given plant species in the local community is often regulated by its natural enemies [81,82,83,84,85,86,87,88]. Thus, the interaction between the invasive plants and their natural enemies, such as herbivores and pathogens, is one of the essential factors influencing the naturalization success and increasing population of invasive plants. Monophagous herbivores and specific pathogens for many invasive plant species are limited in the introduced ranges because of a lack of co-evolutional history between the invasive plants and the pathogens and herbivores that are present {85].

In field surveys in South Florida, *M. micrantha* had a lower diversity of monophagous insect herbivores compared to its native ranges [89]. Similarly, a field survey in southern China yielded only a few herbivore insects, mites, and pathogenic fungi [90]. Many arthropod and pathogen species were selected in field surveys in Malaysia and India as biocontrol agents for *M. micrantha.* However, all of the species selected were polyphagous and/or failed to show sufficient efficacy to control *M. micrantha* [91,92,93]. These observations suggest that natural enemies of *M. micrantha* in its introduced ranges are limited, and the absence of abundant or diverse natural enemies may contribute to the naturalization success of *M. micrantha* in its introduced ranges. In addition, *M. micrantha* has been reported to have anti-fungal pathogen, anti-insect, anti-nematode activity, as reviewed below.

### 3.2. Anti-Fungal Pathogen Activity

Several organic solvent extracts of *M. micrantha* leaves and roots inhibited the growth of four pathogenic fungi, *Fusarium solani* (Mart.) Sacc., *Rhizoctonia solani* J.G.Kühn, *Phytophthora parasitica* Dastur, and *Pythium aphanidermatum* (Edson) Fitzp. The solvents used were methanol, ethyl acetate, acetone, and chloroform. Growth inhibitory activity of chloroform extract was the greatest among these extracts [94]. Five sesquiterpenes, mikanolide, dihydromikanolide, deoxymikanolide, scandenolide, and dihydroscandenolide, were isolated from the chloroform extract as active compounds [94,95]. Among these sesquiterpenes, deoxymikanolide showed the greatest inhibitory activity against these pathogen fungi [96].

Rhizosphere soil of *M. micrantha* inhibited the growth of a pathogenic fungus, *Fusarium graminearum* Schwabe [97]. The pathogenic fungi *Pseudorobillarda*, *Massarina* and *Robillarda* spp. were less abundant in *M. micrantha*-infested soil. The application of dihydromikanolide in the soil also suppressed the abundance of these pathogenic fungi [98]. The pathogenic fungi *Fusarium oxysporum* Schltdl. and *Ralstonia solanacearum* (Smith) Yabuuchi, Kosako, Yano, Hotta & Nishiuchi, and type III pathogenic genes were observed to be less abundant in *M. micrantha* rhizosphere soil. On the other hand, the biocontrol bacteria, *Catenulispora*, *Pseudomonas*, and *Candidatus entotheonella* Schmidt et al., along with polyketide synthase genes, were abundant in *M. micrantha* rhizosphere soil [97,99]. These biocontrol bacteria produce and release antibiotics and polyketides to inhibit the growth of pathogenic fungi. The polyketide synthase genes are involved in synthesizing antibiotics and polyketides in these biocontrol bacteria [100,101]. Such biocontrol bacteria may suppress the abundance of pathogenic fungi, such as *Fusarium oxysporum* and *Ralstonia solanacearum,* through the release of antibiotics and polyketides.

**Figure 3:**
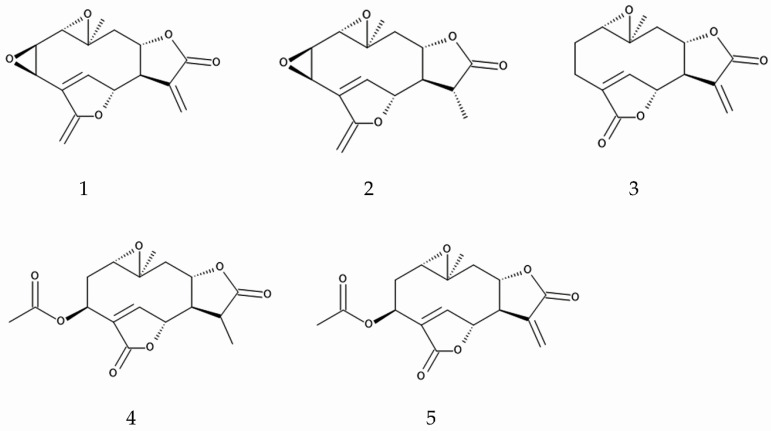
Compounds involved in anti-fungal activity of *M. micrantha.* 1: mikanolide, 2: dihydromikanolide, 3: deoxymikanolide, 4: scandenolide, 5: dihydroscandenolide.

These observations suggest that *M. micrantha* may possess certain compounds involved in anti-fungal activity, such as mikanolide, deoxymikanolide, scandenolide, dihydroscandenolide, and dihydromikanolide, and some of them may be released into its rhizosphere soil through the root exudation and decomposition process of the plant residues (Figure 3). *M. micrantha* may also possess certain compounds involved in the enhancement of bacterial populations used in biocontrol, such as antibiotics and polyketides [97,99]. These compounds may help suppress pathogenic fungi populations, and help increase biocontrol bacteria populations in introduced ranges.

### 3.3. Anti-Insect and Nematode Activity

Alcoholic extracts of *M. micrantha* leaves suppressed the oviposition of a citrus leaf miner (*Phyllocnistis citrella* Stainton) [102]. Methanol extracts of *M. micrantha* leaves suppressed the growth and development of the larvae of a rhinoceros beetle (*Oryctes rhinoceros* L.) [103]. The methanol extracts caused developmental delay and decreased populations of the coconut leaf beetle (*Brontispa longissimi* Gestro) [104]. An essential oil derived from *M. micrantha* caused suppression of oviposition and repellent activity against the diamondback moth (*Plutella xylostella* L.), reducing the time spent by ovipositing moths by as much as 79%. Furthermore, volatile compounds reported as emitted from *M. micrantha*, such as α-terpinene, limonene, and linalool, show oviposition and repellent activity against the diamondback moth at efficacies ranging from 38 to 64% [105].

Parasitic nematodes cause significant injuries and reduce plant vigor [106,107,108]. The abundance of parasitic nematodes *Pratylenchus*, *Hemicycliophora*, and *Longidorus* was lower in *M. micrantha*-infested soil than in the soil of *Perdcaria chinensis* (L.) H.Gross, a native plant species [38].

**Figure 4:**
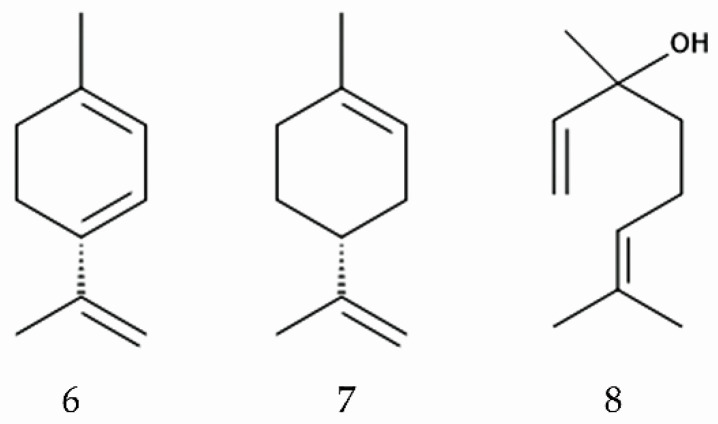
Compounds involved in anti-insect activity of *M. micrantha.* 6: α-terpinene, 7: limonene, 8: linalool.

These observations suggest that *M. micrantha* has anti-insect and anti-nematode activity, and that certain compounds may be involved in the activity (Figure 4). These compounds may contribute to naturalization success and high populations of *M. micrantha* in introduced ranges.

### 3.4. Interaction of M. micrantha with Microbial Organisms

*M. micrantha* infestation has been reported to alter both the soil microbial community and soil chemical characteristics. The infestation of *M. micrantha* produced a significant increase in aerobic bacteria and a simultaneous decrease in anaerobic bacteria in the soil microbial community and soil organic matter, along with increases in pH, total nitrogen, and phosphorus [36,109].

When the residue of *M. micrantha* was mixed with the litter of seven native plant species, *Pinus massoniana* Lamb., *Ficus virens* Aiton, *Litsea glutinosa* (Lour.) C.B.Rob., *Cinnamomus camphora* (L.) J.Presl, *Acacia confusa* Merr., *Schima superba* Gardner & Champ. and *Castanopsis chinensis* (Spreng.). Hence, the decomposition rate of the litter of these native plants was increased [110]. Aqueous extracts of *M. micrantha* increased the litter decomposition rate of *P. massoniana*, *F. virens* and *Acacia richii* A.Gray and the availability of nitrogen in the soil [111,112]. *M. micrantha* infestation accelerated the litter decomposition of native plant species and increased total carbon and nitrate (NO_3_^−^) in the soil [37,113] which can be seen as a legacy effect of such plant invasions [114].

Soil potassium is usually fixed in silicate, and most plants can use only 1–2% of soil potassium [115,116]. However, the potassium-solubilizing bacterium *Burkholderia* spp. was found in *M. micrantha*-infested soil. This bacterium liberates potassium from feldspar, aluminosilicate minerals, and residues of organic matter, and increases potassium solubilization and availability for *M. micrantha* [117]. The abundance of the phosphorus-solubilizing bacteria *Pseudomonas* and *Enterobacter* was higher in *M. micrantha*-infested soil than non-infested soil, and the availability of phosphorus in *M. micrantha*-infested soil was also high [99].

When aqueous leaf extracts of *M. micrantha* were applied to *M. micrantha*-non-infested soil, the nitrate (NO_3_**^−^**) concentration and net nitrification rates in the soil were increased [39]. Nitrification is the oxidation process of ammonia (NH_4_) to nitrate (NO_3_**^−^**) via nitrite (NO_2_**^−^**) involved in the nitrogen cycle. Plant invasions have sometimes been associated with the increasing ammonia-oxidizing bacteria abundance and nitrogen cycling [118]. The extracts of *M. micrantha* also increased ammonia-oxidizing bacterium abundance [39], which suggests that certain compounds in extracts and in *M. micrantha*-infested soil may affect ammonia-oxidizing bacteria activity.

The abundance of the bacteria involved in the nitrification and carbon, phosphorous, and sulfur metabolism in *M. micrantha*-infested soil was higher than in non-infested soil [119]. *M. micrantha* infestation significantly affected the microbial function related to the metabolism for organic matter in soil, resulting in the increasing availability of nitrogen, carbon, phosphorus, and sulfur [120]. When dihydromikanolide was applied to non-infested soil, the densities of the bacteria involved in the nitrification, and carbon, phosphorous and sulfur metabolism were increased [98]. Therefore, the high abundance of these bacteria in *M. micrantha*-infested soil may be caused by dihydromikanolide. Dihydromikanolide may affect the soil microbial population and function and accelerate the release of available nutrients.

These observations suggest that *M. micrantha* may alter the microbial population and function related to carbon, nitrogen, phosphorus, potassium, and sulfur metabolism in its rhizosphere soil by releasing certain compounds into the soil, such as dihydromikanolide, and may increase the nutrient availability such as carbon, nitrogen, phosphorus, potassium, and sulfur. The improved nutrient condition created may contribute to the invasion success of *M. micrantha* in new habitats as seen for many other invasive plants [121].

### 3.5. Allelopathy

Allelopathy is the interaction between donor plant and receiver plant species in the neighboring plant community through certain compounds, defined as allelochemicals. Allelochemicals are produced and released by the donor plant species, and inhibit the germination, growth and/or development of the receiver plant species [122,123,124,125]. The allelopathic potential of the invasive plant species against the native plant species is often reported to be high [126,127,128,129].

Both aqueous extracts and several organic solvent extracts of *M. micrantha* leaves, roots and/or stems suppressed the growth of *Lycopersicon esculentum* Mill. and *Brassica chinensis* L. [130], and the germination and growth of *Eleusine indica* (L.) Gaertn., *Cyperus iria* L. and *Ageratum conyzoides* L. [131], and also *Lagerstroemia indica* L., *Robinia pseudoacacia* L. and *Lolium perenne* L. [132,133,134]. The extracts also suppressed the germination, growth, biomass, chlorophyll contents of *Macrotylama uniflorum* (Lam.) Verdc. [135]. These observations suggest that *M. micrantha* leaves, roots and/or stems may contain certain extractable allelochemicals.

The incorporation of *M. micrantha* leaves and roots into field soil resulted in the suppression of the germination and/or growth of *Eleusine indica*, *Cyperus iria* and *Ageratum conyzoides* [131], *Asystasia intrusa* (Forssk.) Blume, *Chrysopogon aciculatus* (Retz.) Trin. and *Paspalum conjugatum* P.J.Bergius [136], and *Lycopersicon esculentum* Mill. and *Brassica chinensis* (L.) Hanelt [130]. These observations suggest that certain allelochemicals may be released from leaves and roots of *M. micrantha* into rhizosphere soil, and cause growth inhibition. Allelochemicals in leaves and roots may also be released into the soil during their decomposition process.

Three sesquiterpenoids—dihydromikanolide, deoxymikanolide, and 2,3-epoxy-1-hydroxy-4,9-germacradiene-12,8:15,6-diolide—were isolated from the ethanol extracts of *M. micrantha* leaves. These compounds inhibited the growth of *Lactuca sativa* L., *Lolium multiflorum* Lam., *Acacia mangium* Willd., *Eucalyptus robusta* Sm., and *Pinus massoniana* Lamb. Inhibitory activity of these compounds was not significantly different [132]. Twelve thymol derivatives were isolated from the aqueous ethanol extracts of *M. micrantha* roots. Among them, 8,10-dihydroxy-9-benzoyloxythymol and 8,10-dihydroxy-9-(2-methylbutyryloxy) thymol showed the higher inhibitory activity against the germination and shoot growth of *Arabidopsis thaliana* (L.) Heynh [137]. Ent-kaurene diterpene glucoside, β-D-glucopyranosyl-15α-(3-hydroxyl-3-methylbutanoyloxy)-ent-16-kauren-19-oate was also isolated from the aqueous ethanol extracts of *M. micrantha* roots. This compound inhibited the growth of *A. thaliana* [138]. These sesquiterpenoids and ent-kaurene diterpene glucoside may contribute to the growth of inhibitory activity of *M. micrantha* extracts.

Volatiles emitted from *M. micrantha* leaves and flowers suppressed the germination and growth of *Lactuca sativa*, *Chrysanthemum coronarium* L., *Bidens pilosa* L., and *Abutulon theophrasti* Medik. These were identified as α-Terpineol, β-ocimene, β-myrcene, α-pinene and β-caryophyllene [139]. β-Caryophyllene emitted from the leaves of *M. micrantha* also inhibited the germination and growth of *Raphanus sativus* L., *Brassica campestris* L., and *Lactuca sativa* [140]. These volatile compounds increased the malondialdehyde (MDA) content and decreased the activity of superoxide dismutase (SOD) and catalase in these target plant species [139]. These observations suggest that *M. micrantha* may release allelochemicals as volatiles into the neighboring environment.

The aqueous extracts of *M. micrantha* leaves, stems and roots also increased MDA content and decreased the activity of catalase in *Coix lacryma-jobi* L. and *Triticum aestivum* L. [141,142]. Benzoic acid and cinnamic acid were isolated from the aqueous extracts of *M. micrantha* leaves. Both acidic compounds decreased chlorophyll contents and the activity of SOD, and increased MDA content in *Chrysanthemum coronarium* leaves [143].

Because it reacts to reactive oxygen species (ROS) and/or lipoxygenase activity, MDA content acts as a lipid peroxidation marker related to oxidative stress, which plants may experience through allelopathy. Indeed, a significant increase in free MDA in certain plant tissues was observed under oxidative stress conditions [144]. SOD and catalase were reported to be induced under oxidative stress conditions and to convert ROS to molecular oxygen and hydrogen peroxide [145,146,147]. Oxidative stress often causes significant damage to plant cellular components, leading to the interruption of the physiological functions in the plant cells. Thus, oxidative stress is controlled by the antioxidant defense system, and SOD and catalase are involved in the system [148,149]. Considering the observations described above, volatile allelochemicals—α-terpineol, β-ocimene, β-myrcene, α-pinene and β-caryophyllene, benzoic acid, and cinnamic acid—may cause oxidative stress conditions and suppress the activity of the antioxidant enzymes; SOD and catalase, resulting in the interruption of the physiological functions in the target plant cells. Benzoic acid and cinnamic acid were reported to cause a reduction of chlorophyll content in *Chrysanthemum coronarium* leaves [143], which may be one of the symptoms caused by oxidative stress conditions.

**Figure 5:**
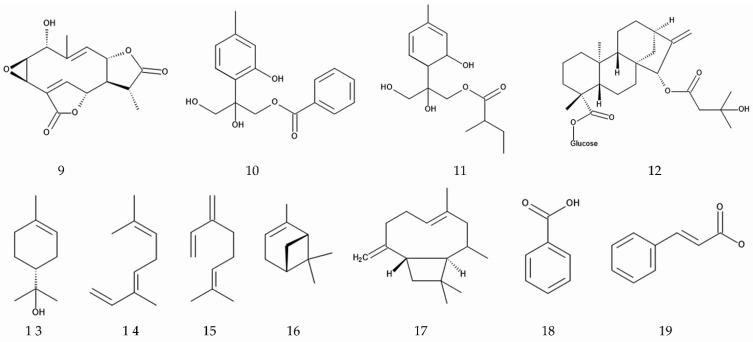
Compounds involved in allelopathic activity of *M. micrantha.* 9: 2,3-epoxy-1-hydroxy-4,9-germacradiene-12,8:15,6-diolide, 10: 8,10-dihydroxy-9-benzoyloxythymol, 11: 8,10-dihydroxy-9-(2-methylbutyryloxy)thymol, 12: β-D-glucopyranosyl-15α-(3-hydroxyl-3-methylbutanoyloxy)-ent-16-kauren-19-oate, 13: α-terpineol, 14: β-ocimene, 15: β-myrcene, 16: α-pinene, 17: β-caryophyllene, 18: benzoic acid, 19: cinnamic acid.

In summary it is clear that *M. micrantha* is allelopathic and produces a variety of allelochemicals (Figure 5). These allelochemicals may be released into the neighboring environment and cause adverse conditions, such as an oxidative stress condition in nearby plants, leading to the interruption of physiological functions and growth suppression. Thus, the allelopathic effects of *M. micrantha* may contribute to the invasion success of *M. micrantha* in new habitats.

## 4. Conclusions

As the evidence we have compiled indicates, *Mikania micrantha* is highly invasive and particularly thrives in disturbed habitats such as forest margins, riparian zones, roadsides, and various agroecosystems. A key aspect of its invasiveness is its rapid growth via a unique physiology, including its efficient photosynthetic capacity [4,49,50]. The stems extend their multiple branches and attach to vegetation, often overtopping tree canopies, reducing the vigor of affected plants [12,28,51,52]. Given this strong competitive ability, *M. micrantha* reduces the populations and biodiversity of native plants in its introduced ranges. Certain crops are more vulnerable to the infestation of *M. micrantha* because of its climbing habit, leading to significant yield loss [27,28,30,68]. These competitive mechanisms are already enough to enable it to frequently dominate invaded communities, but its allelopathic mechanisms make it that much more competitive.

*M. micrantha* exhibits many reproductive traits that enhance its invasiveness, including high levels of seed production [53,54] and seed dispersal via wind or water [53,55,56]. Furthermore, it is also prolific via asexual means [5,28,43,51]. These reproductive parameters promote rapid population growth and are made even more potent when considering the chemical defenses of *M. micrantha*.

The species is a self-incompatible and outcrossing species exhibiting a high degree of genetic diversity within and among populations [57,76]. The various properties related to its genome provide a strong basis for the adaptability of *M. micrantha* [4]. Epigenetics also contributes to the adaptability of *M. micrantha* [77,78]. The high levels of genetic variation exhibited by *M. micrantha* also have implications for its chemical defenses, though these implications have not yet been researched extensively.

Invasion by *M. micrantha* results in a significant increase in aerobic bacteria and a decrease in anaerobic bacteria in the soil microbial community, along with increases in pH, total nitrogen, and phosphorus, and decreases in soil organic matter [36,109]. This invasive plant also alters the function of the microbial community related to carbon, nitrogen, phosphorus, potassium, and sulfur metabolism in its rhizosphere soil through the release of certain compounds. The resulting improved nutrient conditions may well contribute to the invasion success of *M. micrantha* in new habitats.

The interaction between invasive species and their natural enemies, such as pathogens and herbivores, is often key to understanding the successful naturalization of invasive plants. Whether a plant goes beyond simply being introduced to become recognized as a harmful invasive often depends on these relationships. The natural enemies of *M. micrantha* in its introduced ranges are limited, and this absence of natural enemies likely contributes to the naturalization success of *M. micrantha*. This calls for increased research on and deployment of biocontrol agents, accounting for the genetic plasticity and defense systems *M. micrantha* possesses against insect and fungal enemies. In addition, *M. micrantha* releases compounds involved in anti-fungal activity into its rhizosphere soil through the root exudation and decomposition process of the plant residues [94,95,97,99]. *Mikania micrantha* contains a variety of compounds involved in anti-insect and anti-nematode activity [102,103,104,105], but more research is needed to better characterize its defenses against its natural enemies.

*Mikania micrantha* produces allelochemicals, which may be released into the neighboring environment including the rhizosphere soil [137,138,139,140]. Some of these allelochemicals cause an oxidative stress condition in nearby plants [141,142,143]. These allelochemicals interrupt the physiological functions of the receiver plant species, leading the suppression of the germination, growth, and/or regeneration process of the neighboring plant species. Subsequently, *M. micrantha* may monopolize many resources such as light, water and nutrients sought by the local plant community [122,123,124,125]. Competition between invasive plants and native plant species to acquire limited resources is a major determinant of the success of invasive plants in their introduced range. Thus, the allelopathic potential of *M. micrantha* reviewed here may contribute to the invasion success of *M. micrantha* in new habitats.

Phytochemical and pharmacological investigations suggested that *M. micrantha* contains many secondary substances, such as phenolic acids, flavonoids, tannins and terpenoids. Some of these compounds have pharmacological activity [150,151,152,153,154,155]. To this point, many of these compounds have yet to be connected to defense functions against natural enemies, such as insects, pathogen fungi, parasitic nematodes, and other plant competitors via allelopathy. Many secondary substances from invasive plants have been reported to have multiple functions, such as anti-pathogen, anti-herbivore, and allelopathic activity [156,157,158,159,160]. As these compounds may comprise part of the defenses *M. micrantha* against its natural enemies, there is much scope for future research to attempt to discern the potential roles of these compounds in defense. The fact that fewer natural enemies occur in its introduced range also leads to rapid growth and spread of *M. micrantha* in the short term and may well enable the plant to evolve more novel chemical defenses as it continues to form large, genetically diverse populations in its introduced range.

In this review, we have summarized what is known about the five major mechanisms of the invasiveness of *M. micrantha* listed in Table 1: rapid growth, high reproductive capacity, high levels of genetic adaptability, defenses against natural enemies, and allelopathy. Hitherto, considerable research has quantified the first three mechanisms, but relatively little research has been conducted on the defensive capabilities inherent in the secondary chemistry produced by *M. micrantha* that help defend it against insect, fungal and plant enemies. In many cases, the chemicals implicated in potential defense mechanisms have been identified, but the details of the mechanisms still need to be elucidated. What we have documented from the literature should provide a helpful starting point to further understand these defense mechanisms and how weed management strategies can account for them.

## Figures and Tables

**Table 1:** The invasive mechanisms of *Mikania micrantha*.

Characteristic	References
**Growth:** Rapid growth via rapid stem elongation and branching	[4,47,48,49,50]
Efficient photosynthetic capacity	[4,48,49,50]
Ability to smother vegetation, blocking photosynthesis	[11,12,13,28,44,52]
**Reproduction and dispersal:** High capacity for reproduction and dispersal	[7,14]
Production of large numbers of seeds	[53,54,58]
Effective vegetative reproduction	[5,28,43,51]
Dispersal by wind and water	[56,61]
**Adaptability:** High levels of adaptability	[4,60]
High genetic variation	[17,74,75,76]
High epigenetic variation	[77,78]
**Defenses:** A variety of defenses against natural enemies	[96,99,110]
Few natural enemies in introduced range	[89,90]
Anti-fungal, anti-insect and anti-nematode activity	[94,95,96,97,98,99,102,103,104,105]
Allelopathy and allelochemicals	[6,45,134,136]
Germination and growth suppression of neighboring plants	[130,131,132,133,134,135,136,137,138,139,140,141,142,143]
